# Anyone but Me: Unrealistic Optimism, Emotions and Anxiety in the Face of COVID-19 Pandemic

**DOI:** 10.3390/ijerph20010301

**Published:** 2022-12-25

**Authors:** Adrianna Wielgopolan, Maciej Pastwa, Aleksandra Warkocka, Kamil Konrad Imbir

**Affiliations:** Faculty of Psychology, University of Warsaw, 00-927 Warsaw, Poland

**Keywords:** COVID-19, unrealistic optimism, reflective emotions, automatic emotions

## Abstract

The worldwide COVID-19 pandemic (and its consequences, such as lockdown and public health regimes) was a novel and stressful situation for most of people, and, as such, it significantly affected both cognitive and emotional functioning of individuals. In our study, we explored unrealistic optimism bias (the cognitive error giving people a feeling of invulnerability) and any declared preventive behaviours undertaken in order to minimise the risk of contagion. We also measured twelve specific emotions (differing in valence and origin) and the feeling of the anxiety caused by the coronavirus. The results allowed us to confirm the occurrence of unrealistic optimism bias (being significantly stronger for men than women), which correlated negatively with the declared number of preventive behaviours. Unrealistic optimism was also positively correlated with negative automatic emotions and negatively correlated with positive reflective emotions. We created models accounting for the variance of general anxiety, finding significant predictors for both separate groups of younger and older adults (negative emotions, both automatic and reflective; and preventive behaviours). However, there was an effect of positive emotions (both automatic and reflective) having a protective role from the feeling of general anxiety, which was significant for the older group only. Our findings may be a valuable cue for coping with crisis situations.

## 1. Introduction

The outbreak of the COVID-19 pandemic was a great cause of stress and anxiety all over the world. Some authors argued that the stress itself might have lowered immune system defences, thereby, becoming a risk factor for the actual disease (Kim and Su, 2020). Regardless of such consequences, many authors suggested that levels of stress and anxiety rose in the general population to very high levels during the pandemic, and it will take a long time for people to recover from such changes [1,2].

### 1.1. The Context—Beginning of the Pandemic

Our study was conducted in Poland, between 30 March and 29 April, 2020. This was a time when there was a dynamic rise in the number of people diagnosed with COVID-19, from 2055 diagnosed subjects on 30 March to 12,640 diagnosed subjects on 29 April. Studies conducted in other countries during the early stages of the pandemic confirmed a quick rise in feelings of stress and anxiety among the general population, both in places seriously affected by the virus [3,4,5,6,7] and in those where the disease was not such a serious problem [8,9]. Furthermore, in the aforementioned time period, numerous regulations and recommendations were introduced in Poland, including (but not limited to) closing the borders, a ban on public gatherings, limitations on the number of people using public transport, wearing masks outdoors, starting online education for schools and universities, and the closure of parks, boardwalks and beaches (Dz.U. [Journal of Laws] from 2020, item 491, 522; Dz.U. [Journal of Laws] from 2020, item 566, 577; Serwis Rzeczypospolitej Polskiej, 16 April, 2020). These regulations were not novel (and possibly stressful in their own rights), they also created very dynamic and changing conditions. Previous studies showed that the outburst of the COVID-19 pandemic, as well as the consecutive lockdowns, caused people to feel threatened, and to experience higher levels of depressive and anxiety symptoms [3,10,11,12,13,14]. Especially at the beginning, the constantly changing restrictions and recommendations could elicit feelings of uncertainty, fear, worry, anxiety or panic [15], as well as loneliness or frustration (which were especially probable after a long quarantine; Hsing et al., 2020).

### 1.2. Unrealistic Optimism

Certain factors have been found to influence how people perceive threat to health and what actions they take. For instance, while predicting their future, people may have either a tendency to anticipate primarily positive events, somehow beneficial for them (an attitude defined as optimism), or mostly negative events, subjectively assessed as unfortunate (defined as pessimism). It is quite common to think about optimism as the proper approach, which may have a positive impact on one’s wellbeing [16], and even physical health [17]. However, it seems like this is not always the case and definitely not when optimism becomes a cognitive error of judgement.

Generally, optimistic people tend to think about themselves as invulnerable; some misfortunes may happen to others, but definitely not to them [18]. While subjectively lowering the risk of negative events, they also assess positive events as more probable to happen to them than other people [19,20]. Evaluating whether such an optimistic way of thinking is realistic might be difficult for an individual, but on a group basis, it is rather simple. If there is an overall collective tendency to anticipate mostly positive events (so most people believe they are less prone to negative events than others, which is logically impossible), regular optimism turns into a systematic error of judgement, a phenomenon referred to as unrealistic optimism. People assess that, compared to others—, they are less likely to have a car accident [21], develop lung cancer [22], skin cancer [23] or to have a heart attack [24].

Most studies regarding unrealistic optimism consist of asking participants about events they are rather familiar with. People have in mind that car accidents and heart attacks may happen, as these are seen as just parts of everyday life [25] and some people may have even calculated the risk of facing those events (based, for example, on diseases present in one’s family). For that reason, it seemed particularly interesting to study unrealistic optimism bias in a very real, uncommon and threatening situation, such as the coronavirus pandemic, when the whole situation (recommendations, restrictions and dynamics of contagion) changed extremely fast and required a constant cognitive appraisal from people. Previous studies showed that unrealistic optimism may have been present during the early stages of the COVID-19 pandemic among university students, who assessed their chances of contagion as significantly lower than those of other people [19]. Similarly, this phenomenon was visible in manipulation of distance, whereby people assessed the threat of contagion in their own country as significantly lower than the threat in Europe and the whole world [26]. The importance of the occurrence of unrealistic optimism is due to the fact that it may play a crucial role in whether preventive behaviours are undertaken [27]. In a study by Park et al. [28], optimistic bias was negatively related to the perception of the probability of getting infected by coronavirus and, furthermore, to the amount of preventive and informative behaviours undertaken by participants. It seems as if there was simply no point in individuals actively making an effort and trying to avoid getting infected if they thought they would not get infected in the first place. The illusion of invulnerability may especially shape behaviour during a threat.

### 1.3. Emotions

The affective state may have both informational and directive functions for an individual. The informative function allows one to make an appraisal of the current situation, based on the experienced emotional state [29], and constitutes something of a barometer regarding the current situation [30]. Positive emotions are a signal that everything is in order, or at least relatively close to the desired state of affairs, having a significant impact on cognitive structure and organization [31,32,33]. It may also, for example, increase creativity in problem-solving [34]. On the other hand, negative emotions are a clue that there is something wrong. Negative emotions may indicate a possible threat or an obstacle in achieving some goal. Negative affect makes an individual more attentive, alert, and ready to take action [35,36,37].

An important recently proposed dimension allowing one to describe emotions’ diversity as well as explain the mechanisms underlying the formation of emotions is the origin of emotion [38,39]. The origin is composed of two different mechanisms of emotion formation: (1) automatic, based on immediate reactions to stimuli, and (2) reflective, based on careful consideration. The automatically originated emotions are due to the innate or well-trained mechanisms of stimulation evaluation, and lead to immediate emotional reactions, and, thus, should be present when some unexpected situations happen. Emotions which are reflectively originated are due to the cognitive process called appraisal, based on verbalized criteria of what is wrong or good. Thus, they appear after a necessary delay for situation interpretation. Therefore, reflective emotional processes should be considered as long-term emotional reactions to crises, such as a pandemic.

Apart from categorizing emotions by their valence (positive vs. negative) and origin (automatic vs. reflective), they might also be differentiated by functionality [36,37,40], which is very much context-dependent. Emotions may be functional, serving a specific purpose, bringing information, and motivating action; e.g., anxiety in a new, potentially dangerous situation. Afunctional emotions serve no purpose and are useless, usually very low on the activation dimension, e.g., grief or sadness. Dysfunctional emotions have a negative impact on an individual, e.g., phobias or panic [36,41,42].

People may feel various emotions not only because of the situation they are in but also because of their individual differences in emotional functioning. Gray [43] and Lazarus [30] both stressed the role of personality and cognitive processing of emotional arousal in this diversity. Davidson and Begley [44] created a concept of affective style, based on neurobiological findings (lateralization of hemispheres), which indicated that people may differ in the following: how fast they recover from an emotion, how long they stay in an emotional state, how well they detect and distinguish their own and others’ emotions, how fluent they are in controlling and regulating emotions and how sensitive to emotions they are. Thanks to this variety, people may obtain complex and unique patterns of experiencing, understanding and regulating their emotions [45]. It seems that these differences may be especially emphasized in a situation as extreme, unfamiliar, threatening, and uncontrollable as the coronavirus pandemic.

The whole unique pattern of emotional functioning may also be a predictor of experienced distress, i.e., anxiety. Among the Italian population, individuals with cyclothymic and depressive temperaments were at greater risk for experiencing mild and severe distress regarding the pandemic situation [7]. Meanwhile, the fear of the coronavirus itself might have been a significant predictor of behavioural change, such as social distancing, indicating how emotions might have shaped behaviour during the pandemic [46].

### 1.4. Other Factors Influencing Anxiety: Sex and Age

It is believed that women are more endangered by stress and anxiety than men, which may derive from the process of socialisation. As society expects women to show symptoms of anxiety and handle them differently than men (no need to hide the symptoms), women may experience stress and anxiety more consciously and, in consequence, showing more symptoms in research [47]. This seems to be confirmed by studies conducted during the COVID-19 pandemic, in which women also showed more anxiety symptoms than men [7,48].

The results of studies exploring how the pandemic influenced anxiety in different age groups seem inconsistent. Some studies reported straightforward results, where the oldest people showed more symptoms of distress [9,49,50,51], which seems understandable, as seniors were the ones most endangered by the disease itself. Other studies, however, reported that the youngest groups were the ones most concerned about the pandemic and reported most symptoms of distress and anxiety [5,14,50,52]. The reason for this inconsistency may be the mediating role of the availability of information about the spread of the pandemic, administrative regulations and healthcare programmes related to COVID-19. Different age groups tend to choose different sources of information. Younger people tend to rely mostly on information from online media, while older groups mostly follow traditional media. Furthermore, perhaps the different age groups might have different characteristics and mechanisms, which allowed them to deal differently with the situation. A previous study showed, for example, that with age, we improve our emotional well-being, and we may feel and notice a bigger range of emotions [53]. It seems plausible that this variety of emotions, rather than causing one to drown in overwhelming fear, may be responsible for better resilience [54] or even detachment from unpleasant situations [55].

## 2. Aim and Hypotheses

The main aim of our study was to verify the presence of the phenomenon of unrealistic optimism during the situation of the coronavirus pandemic. We also wanted to measure the feeling of anxiety caused by coronavirus (and various facets of this threat, including its controllability, seriousness, realness, and familiarity) and specific emotions experienced by people in this particular situation, such as being protective, and the risk factors regarding feeling of anxiety. We took into consideration not only negative emotions [26,56,57] but also positive ones, expecting them to be especially significant in relation to unrealistic optimism and anxiety.

Firstly, we hypothesised, based on some previous studies (e.g., [19,28], that people would show unrealistic optimism regarding the coronavirus pandemic in indicating their own probability for contagion as significantly lower than a probability of contagion for a similar random person. We also expected that there would be differences in the intensity of unrealistic optimism bias between women and men (with men showing significantly more unrealistic optimism than women), as well as in the number of preventive behaviours (with women undertaking significantly more preventive behaviours than men). We predicted that unrealistic optimism would be negatively correlated with the number of preventive behaviours.

As for emotions, we expected them to be the significant predictors of anxiety related to the coronavirus pandemic, with automatic negative emotions being a risk factor, while positive automatic and reflective emotions, together with negative reflective emotions, being protective factors. This protective effect should be stronger for older participants [14], considering developmental changes in experiencing emotions (especially reflective ones).

## 3. Method

### 3.1. Participants

A total of 703 participants, all volunteers, took part in the study (577 women and 126 men). The participants were all aged between 18 and 72 (*M* = 290.56, *SD* = 100.17). We posted the invitation to take part in the study on the Facebook platform, so the participants were users of Facebook and members of various discussion groups dedicated, for example, to particular physical activities (e.g., yoga practitioners or runners), faculties of Polish universities (e.g., biology students from the University of Gdańsk) or inhabitants of particular districts (e.g., the Białołęka district in Warsaw). The participants did not receive remuneration for taking the survey.

We excluded from analyses participants who declared that they were infected with coronavirus (*N* = 2), as there was no point in asking them about the probability of their infection. We also excluded all of the unfinished surveys (118, most of them being dropped at the very beginning of the survey).

All subjects gave their informed consent for inclusion before they participated in the study. All the procedures which involved human participants were conducted in accordance with the 1964 Helsinki Declaration. The protocol was approved by the Research Ethics Committee of the Faculty of Psychology of University of Warsaw.

### 3.2. Materials

In previous studies, there were at least two ways of measuring unrealistic optimism: comparative (e.g., [58]) and absolute (e.g., [19]) judgements. A comparative judgement consists of one question (usually directly asking participants whether, compared to a random person similar to themselves, they are less, more or equally likely to experience some event). An absolute judgement consists of two items, asking participants separately about their probability of experiencing some event and the probability of a random person similar to themselves experiencing the same event. The latter measure seems more conservative, as it may lead to less biased results (it is more indirect, and the participants may not be as focused on the comparative nature of these questions as in the case of the first method [59]. For this reason, we decided to use the absolute measure technique.

The level of unrealistic optimism was operationalized with two questions: “How probable do you think the situation is in which you will get infected with coronavirus?” and “How probable do you think the situation is in which a random person of your age and physical condition will get infected with coronavirus?”. The questions were presented to participants in randomised order (half of the participants were first asked about themselves, half about a random person) in order to avoid any anchoring effects. In the second question, we specifically wanted participants to refer to a random person similar to themselves by age and physical condition, as age [60] and physical condition (e.g., already existing diseases, obesity) may be risk factors to getting infected and experiencing more severe symptoms [61]. Participants gave answers to both questions by setting a bar on a slider beneath each question; they only saw two ends of dimensions (marked as ‘impossible’ and ‘very probable’) without any visible, separating grid lines of the slider, thus being a very intuitive measurement (the slider was set to record answers from 1 to 100). The default position of the bar on all of the sliders was always in the middle.

Afterwards, participants were asked to indicate which emotions they experienced in the current situation. Although crises such as pandemic outbreak are mostly associated with negative emotional experiences, both negative and positive emotions may play a crucial role during this time [62,63], so, in our study, we wanted to include differently valenced emotions. We decided to focus on twelve specific emotions which may be especially relevant in this situation; they were all categorised by their valence (positive vs. negative) and origin (automatic vs. reflective c.f. [39]. For the negative automatic emotions. we chose **helplessness, frustration, and terror**, each associated with the automatic reaction to the deprivation of control. As for positive automatic emotions, we decided to study **consolation, relaxation and release**, each resulting from the automatic reaction to reduction of deprivation (c.f. [39]). For negative reflective emotions, we asked participants about **sadness, disappointment and contempt**, each resulting from the appraisal of the situation in the context of norms or expectations toward the situation. Lastly, for positive reflective emotions we wanted to study **compassion, hope and joy**, each resulting from an appraisal of the situation, changing one’s perspective or predicting positive perspectives for the future (c.f. [39]). Most of these emotions were already linked to some aspects of the pandemic, such as loneliness, loss of usual daily routine or lower amounts of contact with other people, caused by both the lockdown and sanitary regime [46,64,65,66,67]. In the study, our participants saw the 12 aforementioned emotions presented in randomized order, each with a slider right under the emotion’s name. The sliders were identical to the ones used in unrealistic optimism measurement (no gridlines, default position of the bar in the middle of the slider), while the ends of these scales were labelled as ‘to a small extent’ and ‘to a significant extent’.

The feeling of the anxiety caused by coronavirus was measured by a 7-item scale (with Likert-like answers from 1 to 7), consisting of seven questions created for the of this study; Cronbach’s α = 0.79. The first two positions asked about how real the threat of coronavirus seemed (Cronbach’s α = 0.73), another two were related to the controllability of the threat (Cronbach’s α = 0.61) and two more applied to the seriousness of it (Cronbach’s α = 0.86). The last item was about the familiarity of the threat. For the exact items of the questionnaire, see Supplementary Materials 1 (English version) or Supplementary Materials 2 (Polish version).

### 3.3. Procedure

Participants were recruited to the study via Facebook, getting the link to the study in the form of an online questionnaire created on the Qualtrics platform either by public post or via private message. At the beginning of completing the study, participants were informed about the estimated duration of it (about five to seven minutes), the affiliation of the researchers and the general subject of the study (in order to minimise any possible biases, it said only that the survey would be about emotions and behaviours connected to the pandemic situation in Poland). They were also informed that the study was anonymous and voluntary, and that they could stop filling out the survey at any time.

After this initial information, participants answered questions about unrealistic optimism, emotions and anxiety caused by the coronavirus. They were also asked to answer one open-ended question about their behaviour (“*List behaviours which you undertake in order to minimise the risk of getting infected with coronavirus, e.g., washing your hands more often*”). Next, they gave some demographic information about themselves (including age, gender and their level of education). We also asked “*Have you been infected with coronavirus?*”, with a simple ‘Yes’ or ‘No’ as the answer option, to exclude those who answered ‘Yes’. At the end, participants were debriefed about the subject of the study (with a very short definition of unrealistic optimism) and they were given the e-mail address of one of the authors in case they had any questions or doubts. After that, they were thanked for their participation in the study.

## 4. Results

### 4.1. Unrealistic Optimism

To test for the occurrence of the phenomenon of unrealistic optimism, we analysed scores from both questions about probability of infection (about participants themselves and random persons) with the Wilcoxon signed-rank test, as the data did not meet the normality of distribution assumption. Participants evaluated the probability of getting infected with coronavirus (*M* = 62, *SD* = 270.32) as significantly lower than for a random person similar to themselves (*M* = 660.74, *SD* = 270.10), *Z* = −50.92, *p* < 0.001, *η*^2^ = 120.09.

The measure of unrealistic optimism was computed by subtracting the probability of the question about the individual from the probability about a random person. The unrealistic optimism scores were then analysed in order to check for gender differences, using the Mann–Whitney U nonparametric test (because of the large number disproportion between groups). We found a significant difference, indicating that men (*M* = −100.56, *SD* = 240.95) showed more unrealistic optimism than women (*M* = −30.50, *SD* = 200.5); *U* = 29,576, *p* = 0.001, *d* = 00.25.

There was also a significant positive correlation (Spearman’s rho) between unrealistic optimism and negative automatic emotions (*r_s_* = 0.09, *p* = 0.02) and insignificant negative correlation between unrealistic optimism and positive automatic emotions (*r_s_* = −0.06, *p* = 0.13). For the reflective emotions, there was significant correlation for positive ones: *r_s_* = −0.11, *p* = 0.005), and insignificant *r_s_* = 0.05, *p* = 0.18 for negative reflective emotions.

We also used a univariate ANOVA in order to check whether participants with different education levels differed in level of unrealistic optimism. However, there were no differences in levels of unrealistic optimism between people with different levels of education (primary, secondary, high school education and higher education); *F*(3, 699) = 00.76, *p* = 0.52.

### 4.2. Preventive Behaviours

The answers from an open-ended question about behaviours undertaken in order to minimise the risk of getting infected with coronavirus were coded by three independent competent judges. All of them were students of 4th or 5th year psychology, did not take part in the actual study and had no knowledge of the study design or the hypotheses. They were asked to score each answer by counting the number of separate behaviours declared by participants (e.g., “*I wear a mask in public places*”, “*I try to stay at home*”, “*I wash my hands more often than usual*”). Agreement among judges was checked using Kendall’s coefficient of concordance; if it was lower than 1, the score given by the majority (2 out of 3 judges) was kept. Overall, correlations between all judges’ scores were strong (*r_s_* = 0.93, *p* < 0.001). We also checked the intra-class correlation coefficient in a two-way random effect model: *κ* = 0.89, *p* < 0.001. Answers “*I do nothing special/I do everything as usual*” were coded as 0; two answers “*I do only what the government says is necessary*” were coded as 30.5 (which was the general mean score). There was a significant difference between men and women; women reported significantly more behaviours (*M* = 30.75, *SD* = 20.43) than men (*M* = 20.85, *SD* = 10.94), *U* = 27,644, *p* < 0.001, *d* = 00.32.

### 4.3. General Anxiety

Firstly, we wanted to check for the gender differences in the feeling of anxiety caused by Coronavirus. For this, a nonparametric Mann–Whitney U test was conducted (taking into account differences in numerical amount between men and women and non-normal distribution of variables). There were no significant differences in feeling of general anxiety between women (*M* = 40.14, *SD* = 00.04) and men (*M* = 30.95, *SD* = 00.09), *U* = 32,362, *p* = 0.053. There was, however, significant difference in feeling of realness of the threat; namely, women assessed it to be more real (*M* = 90.45, *SD* = 30.20), than men (*M* = 80.72, *SD* = 30.32), *U* = 31362, *p* < 0.05, *d* = 00.18.

After that, we decided to conduct a regression analysis and check whether we may find significant predictors of the general anxiety. In the first model, the most basic one, which we created, with only gender and age as predictors, age was a significant predictor (unstandardised *B* = 00.013, *β* = 00.13, *SE* = 00.004, *t* = 30.37, *p* = 0.001) of feeling of a general anxiety; *R* = 00.14, *R*^2^ = 00.02, *F*(2, 702) = 70.43, *p* < 0.001.

Furthermore, we wanted to see how all our variables may explain the variance of the feeling of anxiety; therefore, in the second model, we put as predictors all four clusters of emotions (positive automatic, negative automatic, positive reflective and negative reflective emotions), unrealistic optimism, number of preventive behaviours, and age. The obtained model was significant and it accounted for 220.7% of the variance in feeling of general anxiety: *R* = 00.49, *R*^2^ = 0. 23, *F*(7, 702) = 300.47, *p* < 0.001. The significant predictors were negative automatic emotions, negative reflective emotions, unrealistic optimism, age, and number of behaviours. Positive automatic and positive reflective emotions were both insignificant predictors (Table 1).

Taking into consideration the large age range in our study (18–72), we decided, finally, to analyse data in two subgroups: young adults, 18-39 years old (*N* = 561; *M* = 250.23, *SD* = 50.18), and older adults, 40–72 years old (*N* = 140; *M* = 460.80, *SD* = 60.10). As before, for each group we used general anxiety as a dependent variable in the model and four emotional clusters, unrealistic optimism and number of preventive behaviours, as predictors. The model for the group of young adults was significant and it predicted 180.40% of the variance of general anxiety; *R* = 00.44, *R^2^* = 00.19, *F*(6, 561) = 220.03, *p* < 0.001. Significant predictors were negative automatic and negative reflective emotions, as well as unrealistic optimism, and number of behaviours.

The model for the group of older adults was significant as well, accounting for 32.6% of the general anxiety’s variance; *R* = 00.65, *R^2^* = 00.40, *F*(6, 140) = 160.40, *p* < 0.001. Negative automatic emotions, negative reflective emotions, and number of behaviours were again significant predictors. However, in contrast to the model for younger adults, in this model positive automatic and positive reflective emotions were significant protective factors, whereas unrealistic optimism was not significant at all (to compare the two regression models, see Table 2).

## 5. Discussion

### 5.1. Unrealistic Optimism

The obtained results allowed us to confirm the general presence of an unrealistic optimism phenomenon regarding the coronavirus pandemic. In line with some previous studies (e.g., [19]), people did assess their own probability of getting infected as significantly lower than those of somebody else. This effect was also stronger for men than for women, reflecting some gender differences, possibly caused by the differences in the socialisation process among women and men [47]. There were no differences in unrealistic optimism between groups with different levels of education, therefore confirming the commonness of said effect and, overall, the frequent illusion of invulnerability. However, as we saw later in the regression analyses, whilst unrealistic optimism was a significant risk factor for younger adults, it was insignificant for older adults. If this was an effect independent of level of education, the effect might weaken with age, since, as we get older, we apparently lose the feeling of being immortal.

Remarkably, unrealistic optimism was also correlated with negative automatic emotions; the higher the level of unrealistic optimism, the higher the level of the negative automatic emotions. However, there was no significant correlation for positive automatic emotions. On the contrary, unrealistic optimism was significantly and negatively correlated with positive reflective emotions, but not with negative reflective emotions. It seems that there is a possibility that unrealistic optimism, besides being a cognitive error of judgement, may be somehow linked to the origin of emotions and individual patterns of emotional functioning. Perhaps negative automatic and positive reflective emotions might play the most important roles in this phenomenon, being, respectively, a risk factor and a buffer.

### 5.2. Anxiety and Emotions

We did not find any difference between males and females in the anxiety evoked by Coronavirus, in contradiction to previous findings [7,47,48]. In the early phase of the pandemic, the level of anxiety rose immediately within the whole society, so it is difficult to track differences in it. However, women appraised the danger of COVID-19 as more factual than men and they also undertook more preventive behaviours. This is an interesting finding that corresponds with studies reporting that women may feel more threatened by the virus than men [7,48].

The results of the study show the protective role of reflective emotions against the anxiety caused by Coronavirus. Experiencing emotions related to more complex, social situations is related to generally perceiving the situation in a more complex context [38,68], which leads to reducing the feeling that COVID-19 is a direct threat to ones’ health. Negative emotions, which were socially acceptable and appropriate during the outbreak of the pandemic, when derived from the automatic system, promoted a focus on the threat of the disease. We concluded that when the emotions were, instead, derived from the systematic mind, they promoted a focus on the pandemic as a situation dangerous not only to oneself, but to the whole society. Emotions of disappointment and contempt are especially related to social judgement, and can be perceived as relatable to the ongoing situation and an indicator of positioning the situation in the social context. Unrealistic optimism was not protective against anxiety, it was a factor increasing it, which supports this reasoning, thinking about oneself as more resistant than others means that one is actually focusing on one’s health (optimism correlating positively with the number of preventive behaviours), while reflective emotions promote perceiving the social complexity of the situation. This result is in congruence with previously mentioned studies that suggested that certain personality traits reduced the anxiety caused by the pandemic [7].

Age in the whole group of participants was a risk factor for anxiety, which is congruent with the results of some studies [9,49,50,51]. As mentioned in the introduction, this may be related to the quality of information people obtained from the media [69], as well as the media coverage of the pandemic being a threat mostly to older people. When we explored the results within the groups of younger and older participants, the structure of the protective and risk factors was similar, with one main exception, positive emotions, both automatic and reflective, turned out to be protective against the anxiety only in the group of older adults. As the protective role of positive emotions is not surprising, the result suggests that younger people did not experience these emotions, being overwhelmed by the negative ones. Older adults are more emotionally developed, and, therefore, experience a wider span of emotions. As we mentioned before, some studies showed that older adults, even in stressful situations, have the capability to feel and notice some positive emotions [70] and show greater resilience to stressful situations, including the pandemic [14,54]. We showed that this effect appeared regardless of the origin of emotions (automatic or reflective) and it could be extended to the context of the beginning of the pandemic in Poland.

### 5.3. Limitations

As for the limitations of this study, the time of collecting responses may be one of them. It was almost a month and during this time the situation changed several times. The Polish government sequentially introduced various sets of restrictions and recommendations and then, gradually, withdrew them. However, it seems that there still might be particular specificity of this situation being new, potentially threatening, interfering with daily routine, extremely dynamic and full of uncertainty, and, therefore, similar for all of the respondents.

Another limitation of our study might be the fact that we decided to study very particular emotions, so our results can be interpreted only in relation to these twelve emotions without further generalisation. Nonetheless, the reason behind this decision was that the emotions chosen were all relevant to the pandemic situation, and, thus interesting to measure. The diversity of the chosen emotions did also allow us to categorise them by their valence and origin. That was especially important in such a complex situation as the Coronavirus pandemic. It seemed very intuitive that the pandemic caused unpleasant emotional distress and heightened the levels of negative automatic emotions. However, as it turned out, positive and reflective emotions may play at least an equally important role in everyday functioning.

Furthermore, the characteristics of our sample should be taken into consideration. We only studied volunteers, and, thus, people who not only had access to the Internet and Facebook accounts to see our invitation in the study, but also were interested enough to take part in it. Therefore, we studied people who were willing to click on the link with the survey about the pandemic, so were probably not only aware of the pandemic, but also acknowledged its importance. Moreover, it may be seen that while we did obtain participants in different age groups, we had more young adults than old and we gathered much more data from women. It is crucial to remember the homogeneity of our sample as a limitation of our study and a potential obstacle in generalising the results into the entire population.

Lastly, it is important to keep in mind that we should not draw any causal conclusions from the regression models that we presented. Our design was correlational and we only obtained the one measurement. Future studies could employ more experimental schemas and check how eliciting different emotions (e.g., by the memories of a particular affect) could change the functioning in a demanding, potentially stressful situation.

### 5.4. Conclusions

The presented results show that the phenomenon of unrealistic optimism was present during the beginning of the COVID-19 pandemic in Poland, consistent with other studies [19,26]. Furthermore, we broadened the previous findings by showing how unrealistic optimism might be connected with different types and emotions (especially negative automatic and positive reflective ones), showing how our affective experiences may be linked to this cognitive bias. Our results may suggest, although needing further validation, that unrealistic optimism has something in common with the emotions; and, as such, may be susceptible to some emotional regulation techniques. Perhaps working on emotional well-being could also affect unrealistic optimism and make people more realistic about a situation.

We also showed the importance of positive emotions as significant buffers in the regression models for older (but not younger) adults. Perhaps these results could constitute a cue for managing people and taking care about their well-being in stressful situations. While the protective role of positive emotions is already well known [71,72], it is important to remember that not everybody is able to experience positive emotions in such a situation. However, perceiving the situation in a more reflective, systematic way seems to be a possibility for all age groups to reduce anxiety and it might be taught and trained in times of need.

We showed the characteristics of functioning, in terms of unrealistic optimism, emotions, and anxiety, in the novel and stressful situation of the COVID-19 pandemic outburst. The results regarding factors protective against the virus may be useful in planning interventions and therapy in situations of acute anxiety and other disorders caused by the stressful situation of the pandemic.

## Figures and Tables

**Table 1:** Regression (for whole group) with general anxiety as a dependent variable. The asterisks symbolize the level of statistical significance: **—< 0.01, ***—< 0.001.

	*B*	*β*	*SE*	*t*
Negative automatic emotions	0.007	0.49	0.001	100.61 ***
Positive automatic emotions	−0.001	−0.04	0.001	−10.01
Negative reflective emotions	−0.003	−0.18	0.001	−40.07 ***
Positive reflective emotions	−0.001	−0.03	0.001	−0.72
Unrealistic optimism	0.005	0.10	0.002	20.84 **
Number of preventive behaviours	0.05	0.13	0.02	30.73 ***
Age	0.02	0.15	0.003	40.45 ***

**Table 2:** Regressions for dependent variables of general anxiety with participants divided into two groups: young adults and older adults. The asterisks symbolize the level of statistical significance: *—< 0.05, **—< 0.01, ***—< 0.001.

	Group of Young Adults				Group of Older Adults			
Predictors	*B*	*β*	*SE*	*t*	*B*	*β*	*SE*	*t*
Negative automatic emotions	0.007	0.46	0.001	80.68 ***	0.007	0.48	0.001	40.93 ***
Positive automatic emotions	<0.001	−0.02	0.001	−0.41	−0.003	−0.18	0.001	−20.22 *
Negative reflective emotions	−0.002	−0.14	0.001	−20.71 **	−0.004	−0.21	0.002	−20.43 *
Positive reflective emotions	0.001	0.07	0.001	10.59	−0.005	−0.26	0.001	−30.45 ***
Unrealistic optimism	0.005	0.12	0.002	30.06 **	−0.001	−0.02	0.005	−0.28
Number of preventive behaviours	0.05	0.02	0.02	20.37 *	0.06	0.18	0.02	20.69 **

## Data Availability

Data are available in a publicly accessible FigShare at https://doi.org/100.6084/m9.figshare0.21202577.v1 (accessed on 18 December 2022).

## Supplementary Materials

The following supporting information can be downloaded at: https://www.mdpi.com/article/10.3390/ijerph20010301/s1. Supplementary File S1: English version of the questionnaire; Supplementary File S2: Polish version of the questionnaire.
