# Supplementary material for: Anyone but Me: Unrealistic Optimism, Emotions and Anxiety in the Face of COVID-19 Pandemic
Source: Int J Environ Res Public Health. 2022 Dec 25;20(1):301. doi: 10.3390/ijerph20010301 (PMC9819969; doi:10.3390/ijerph20010301)
Supplement: Supplementary file 1 [file ijerph-20-00301-s001.zip › Supplementary Materials 1.pdf]

Supplementary File S1 (English version of the questionnaire)

Hi,

we are a team of researchers from the Faculty of Psychology at the University of Warsaw. We would like to invite you to our study about emotions and behaviors related to the situation of coronavirus pandemic in Poland. The study is anonymous and voluntary (you may stop completing it at any moment). Data gathered in the process of investigation will be confidential, analyzed on a group level and used for research purposes only.

By clicking the "next" button, you agree to take part in the study.

Indicate on a scale below how probable do you think is this situation:

I will get infected with coronavirus.

1————————————————————————————————100

Impossible Very probable

A random person in my age and physical condition will get infected with coronavirus.

1————————————————————————————————100

Impossible Very probable

To what extend during the pandemic you feel:

helplessness

To a small extent To a significant extent

1————————————————————————————————100

frustration

To a small extent To a significant extent

1——————————————————————————————100

terror

To a small extent To a significant extent

1——————————————————————————————100

consolation

To a small extent To a significant extent

1——————————————————————————————100

relaxation

To a small extent To a significant extent

1——————————————————————————————100

release

To a small extent To a significant extent

1——————————————————————————————100

disappointment

To a small extent To a significant extent

1——————————————————————————————100

sadness

To a small extent To a significant extent

1————————————————————————————100

contempt

To a small extent To a significant extent

1————————————————————————————100

compassion

To a small extent To a significant extent

1————————————————————————————100

hope

To a small extent To a significant extent

1————————————————————————————100

joy

To a small extent To a significant extent

1————————————————————————————100

Please – using a scale from 1 to 7, where 1 mean „I completely disagree" and 7 – "I totally agree" – indicate to what extend do you agree with each sentence below.

1. Getting infected with coronavirus (causing COVID-19 disease) is a real threat to me.

I completely disagree I totally agree

1——————2——————3——————4——————5——————6——————7

2. I am worried about getting infected with coronavirus.

I completely disagree I totally agree

1——————2——————3——————4——————5——————6——————7

3. It's up to me whether I will get infected with coronavirus.

I completely disagree I totally agree

1——————2——————3——————4——————5——————6——————7

4. I know exactly what to do in order to not get infected with coronavirus.

I completely disagree I totally agree

1——————2——————3——————4——————5——————6——————7

5. I feel that getting infected with coronavirus is not a serious threat to my health.

I completely disagree I totally agree

1——————2——————3——————4——————5——————6——————7

6. I feel that getting infected with coronavirus is not a serious threat to my life.

I completely disagree I totally agree

1——————2——————3——————4——————5——————6——————7

7. Coronavirus is a familiar threat for us.

I completely disagree I totally agree

1——————2——————3——————4——————5——————6——————7

List behaviors which you undertake in order to minimise the risk of getting infected with coronavirus, e.g., washing your hands more often.

Your gender:

Woman

Man

Other

Your age (in years):

__

The highest grade or level of school you have completed or the highest degree you have received:

Primary/middle school

Vocational

Secondary education

Post-secondary education

Have you been infected with coronavirus?

Yes

No

Thank you for completing our survey!

Our goal was to measure emotions felt in the situation of coronavirus pandemic. We also wanted to study the phenomenon of unrealistic optimism, which is a feeling of invulnerability when we have an illusion that something bad simply cannot happen to us. It was interesting for us to see whether this phenomenon might have occurred in the pandemic situation as well.

If you have any questions regarding the study or you would like to see its results, please contact us via e-mail: a.wielgopolan2@student.uw.edu.pl
Thank you again for the participation!
