# Supplementary material for: Anyone but Me: Unrealistic Optimism, Emotions and Anxiety in the Face of COVID-19 Pandemic
Source: Int J Environ Res Public Health. 2022 Dec 25;20(1):301. doi: 10.3390/ijerph20010301 (PMC9819969; doi:10.3390/ijerph20010301)
Supplement: Supplementary file 1 [file ijerph-20-00301-s001.zip › Supplementary Materials 2.pdf]

Supplementary File S2 (Polish version of the questionnaire)

Dzień dobry,
jesteśmy grupą badaczy z Wydziału Psychologii Uniwersytetu Warszawskiego. Przeprowadzamy badanie dotyczące doświadczanych emocji i zachowań w związku z epidemią koronawirusa w Polsce. Udział w badaniu jest zupełnie anonimowy i dobrowolny (można przerwać wypełnianie w każdej chwili). Dane będą analizowane wyłącznie zbiorczo i wykorzystane tylko w celach naukowych.

Klikając przycisk „dalej", wyrażasz zgodę na udział w badaniu.

Zaznacz na poniższej skali, jak bardzo prawdopodobna wydaje Ci się opisana sytuacja:

Mogę zarazić się koronawirusem.

1———————————————————————————————100

Zupełnie nieprawdopodobne Bardzo prawdopodobne

Przeciętna osoba w moim wieku i kondycji fizycznej może zarazić się koronawirusem.

1———————————————————————————————100

Zupełnie nieprawdopodobne Bardzo prawdopodobne

Zaznacz, w jakim stopniu doświadczasz poniższych emocji w obecnej sytuacji.

bezradność

w niewielkim stopniu w znaczącym stopniu

1———————————————————————————————————100

frustracja

w niewielkim stopniu w znaczącym stopniu

1———————————————————————————————————100

przerażenie

w niewielkim stopniu w znaczącym stopniu

1———————————————————————————————————100

zasmucenie

w niewielkim stopniu w znaczącym stopniu

1———————————————————————————————————100

rozczarowanie

w niewielkim stopniu w znaczącym stopniu

1——————————————————————————100

pogarda

w niewielkim stopniu w znaczącym stopniu

1——————————————————————————100

ukojenie

w niewielkim stopniu w znaczącym stopniu

1——————————————————————————100

odprężenie

w niewielkim stopniu w znaczącym stopniu

1——————————————————————————100

wyzwolenie

w niewielkim stopniu w znaczącym stopniu

1——————————————————————————100

współczucie

w niewielkim stopniu w znaczącym stopniu

1——————————————————————————100

nadzieja

w niewielkim stopniu w znaczącym stopniu

1——————————————————————————100

szczęście

w niewielkim stopniu w znaczącym stopniu

1——————————————————————————100

Proszę określić, w jakim stopniu zgadzasz się z następującym stwierdzeniem. Proszę użyć skali od 1 do 7, gdzie 1 oznacza, "zdecydowanie się nie zgadzam", a 7 – "zdecydowanie się zgadzam".

1. Zarażenie się koronawirusem (powodującym chorobę COVID – 19) jest dla mnie realnym zagrożeniem.

1—————2—————3—————4—————5—————6—————7

zdecydowanie nie zgadzam się zdecydowanie się zgadzam

2. Martwię się możliwością zarażenia się koronawirusem.

1—————2—————3—————4—————5—————6—————7

zdecydowanie nie zgadzam się zdecydowanie się zgadzam

3. Mam duży wpływ na to, czy zarażę się koronawirusem.

1——————2——————3——————4——————5——————6——————7

zdecydowanie nie zgadzam się zdecydowanie się zgadzam

4. Dobrze wiem, co robić, żeby nie zarazić się koronawirusem.

1——————2——————3——————4——————5——————6——————7

zdecydowanie nie zgadzam się zdecydowanie się zgadzam

5. Mam poczucie, że koronawirus nie stanowi poważnego zagrożenia dla mojego zdrowia.

1——————2——————3——————4——————5——————6——————7

zdecydowanie nie zgadzam się zdecydowanie się zgadzam

6. Mam poczucie, że koronawirus nie stanowi poważnego zagrożenia dla mojego życia.

1——————2——————3——————4——————5——————6——————7

zdecydowanie nie zgadzam się zdecydowanie się zgadzam

7. Koronawirus jest zagrożeniem, z którym wiemy, jak sobie poradzić.

1——————2——————3——————4——————5——————6——————7

zdecydowanie nie zgadzam się zdecydowanie się zgadzam

Wymień działania, które podejmujesz w związku z pandemią koronawirusa, żeby ochronić się przed zarażeniem (np. częstsze mycie rąk):

____

Płeć:

Kobieta

Mężczyzna

Inna

Wiek:

___

Wykształcenie:

Podstawowe

Gimnazjalne

Średnie

Wyższe

Czy byłeś/byłaś zarażony/zarażona koronawirusem?

Tak

Nie

Dziękujemy za wypełnienie ankiety!

Celem badania był pomiar emocji odczuwanych w związku z sytuacją pandemii. Chcieliśmy również zbadać zjawisko nierealistycznego optymizmu – złudzenie, że jesteśmy nietykalni i złe rzeczy zwyczajnie nie mogą nam się przytrafić. Interesowało nas, czy to zjawisko może mieć miejsce w kontekście pandemii koronawirusa.

Jeśli masz jakiekolwiek pytania odnośnie procedury badawczej lub chciałbyś/chciałabyś poznać wyniki, prosimy o kontakt mailowy: a.wielgopolan2@student.uw.edu.pl

Jeszcze raz dziękujemy za udział w badaniu!
